# Characterization of Course and Terrain and Their Effect on Skier Speed in World Cup Alpine Ski Racing

**DOI:** 10.1371/journal.pone.0118119

**Published:** 2015-03-11

**Authors:** Matthias Gilgien, Philip Crivelli, Jörg Spörri, Josef Kröll, Erich Müller

**Affiliations:** 1 Norwegian School of Sport Sciences, Department of Physical Performance, Oslo, Norway; 2 University of Salzburg, Department of Sport Science and Kinesiology, Hallein-Rif, Austria; 3 WSL—Institute for Snow and Avalanche Research SLF, Group for Snowsports, Davos, Switzerland; University of Rome, ITALY

## Abstract

World Cup (WC) alpine ski racing consists of four main competition disciplines (slalom, giant slalom, super-G and downhill), each with specific course and terrain characteristics. The International Ski Federation (FIS) has regulated course length, altitude drop from start to finish and course setting in order to specify the characteristics of the respective competition disciplines and to control performance and injury-related aspects. However to date, no detailed data on course setting and its adaptation to terrain is available. It is also unknown how course and terrain characteristics influence skier speed. Therefore, the aim of the study was to characterize course setting, terrain geomorphology and their relationship to speed in male WC giant slalom, super-G and downhill. The study revealed that terrain was flatter in downhill compared to the other disciplines. In all disciplines, variability in horizontal gate distance (gate offset) was larger than in gate distance (linear distance from gate to gate). In giant slalom the horizontal gate distance increased with terrain inclination, while super-G and downhill did not show such a connection. In giant slalom and super-G, there was a slight trend towards shorter gate distances as the steepness of the terrain increased. Gates were usually set close to terrain transitions in all three disciplines. Downhill had a larger proportion of extreme terrain inclination changes along the skier trajectory per unit time skiing than the other disciplines. Skier speed decreased with increasing steepness of terrain in all disciplines except for downhill. In steep terrain, speed was found to be controllable by increased horizontal gate distances in giant slalom and by shorter gate distances in giant slalom and super-G. Across the disciplines skier speed was largely explained by course setting and terrain inclination in a multiple linear model.

## Introduction

Alpine skiing is a well-recognized recreational and competition sport. Alpine ski racing has been part of the Olympic program since the beginning of the modern winter Olympic games. Races are held on natural slopes and are organized in four main competition disciplines: Slalom (SL), giant slalom (GS), super-G (SG) and downhill (DH). Each discipline has its own specific course and terrain characteristics. The International Skiing Federation (FIS) has established course regulations to make sure these characteristics are maintained in races organized by FIS. FIS regulations specify, for example, a range of altitude differences between start and finish for each discipline. For SL, GS and SG the regulations set a range of direction changes as a function of the altitude difference between start and finish. The minimum linear distance between gates must be 10m for GS and 25m for SG. However, all of these regulations define only certain boundaries, not restricted values. Furthermore, the aforementioned measures do not provide a complete understanding of the course setting and terrain geomorphology, which is essential with regard to various safety issues. Course setting is not only dependent on the linear distance between gates, but also on the horizontal distance by which two successive gates are set out of line. However, the mean terrain inclination between start and finish—obtained from calculations based on course length and vertical drop from the organizers’ data—does not provide a full understanding of the terrain geomorphologic characteristics of courses, and does not provide an understanding of how course setters adapt courses to the given terrain geomorphologic conditions.

Due to the fact that every third WC athlete suffers an injury each season and every 6^th^ athletes suffers a severe injury each season, ensuring athletes’ safety is a major concern in competitive alpine skiing [1,2]. Therefore, FIS and a worldwide network of research groups work together on the identification of injury risk factors [3–9], injury mechanisms [10–13] and the implementation of measures [14,15] to reduce injury risk [3–7,10,12,14–17]. This research has recently revealed the following aspects: 1) practitioners and experts acknowledge that course setting has a significant impact on speed control and injury prevention [3]; 2) course setting influences different mechanical parameters related to injury risk [8,16]; 3) in SL and GS, speed is influenced by variations in course setting [16,18] and terrain inclination [19]; 4) skier biomechanical injury risk factors are significantly different between the disciplines [7]. Based on these considerations, it seems likely that course and terrain characteristics might be linked to injury risk in all disciplines. However, no study has yet investigated these characteristics and their effect on skiers’ speeds during WC competitions.

Therefore, the current study investigated: 1) the geomorphologic characteristics of the slopes; 2) how courses were set; 3) how courses were adapted to the given terrain geomorphology; 4) how course setting and terrain characteristics varied within and between the competition disciplines, 5) how skiers were exposed to terrain features and 6) whether skier speed was governed by course setting and terrain inclination within and across the disciplines (GS, SG and DH).

## Methods

### Study design

**Fig 1:**
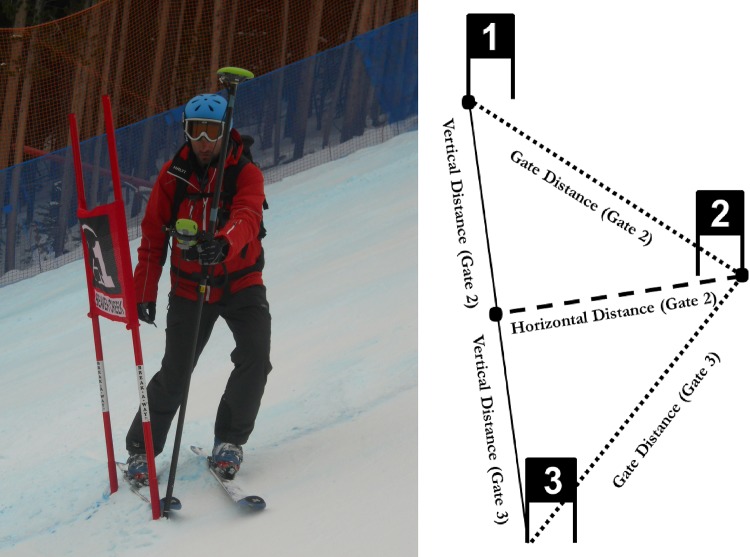
Left: Illustration of the gate position capture. Right: Illustration of the course setting characteristics. Course setting was characterized by gate distance, horizontal gate distance and vertical gate distance.

Course setting and terrain characteristics of male World Cup alpine skiing races were captured in the World Cup seasons 2010/11 and 2011/12. Seven male World Cup GS races—in total 14 runs—(Sölden (twice), Beaver Creek, Adelboden (twice), Hinterstoder, Crans Montana), 5 super-G (SG) races—in total 4 runs—(Kitzbühel, Hinterstoder, Crans-Montana (twice)) and 4 downhill (DH) races (Lake Louise, Beaver Creek, Wengen, Kitzbühel) were captured using static differential global navigation satellite systems (dGNSS). For each race one forerunner was equipped with a dynamic dGNSS to measure skier trajectory. This study was approved by the Ethics Committee of the Department of Sport Science and Kinesiology at the University of Salzburg and athletes signed written consents as approved by the ethics committee (approval number: EC_NR. 2010_03). The International Ski Federation (FIS) and race organizers gave permission to conduct the field study. The individual in Fig. 1 gave written informed consent (as outlined in PLOS consent form) for this image to be published. Other permissions were not required.

### Data collection

#### Digital terrain model and course setting

The terrain geomorphology and course setting were captured using static differential global satellite systems (dGNSS) (Alpha-G3T receivers with GrAnt-G3T antenna (Javad, San Jose, CA, USA) and a Leica TPS 1230+ (Leica Geosystems AG, Heerbrugg, Switzerland)) with the dGNSS antenna mounted on top of a 2m-long pole. The measured antenna position was adjusted for the length of the pole to compute the position of the pole tip (Fig. 1, left side). Differential position solutions were computed in real time and/or in post-processing, applying the post-processing software Justin (Javad, San Jose, CA, USA) and LGO (Leica Geosystems AG, Heerbrugg, Switzerland). The snow surface geomorphologies of the World Cup race slopes were captured during the days directly before the races. The spacing between captured snow surface points depended on the terrain characteristics. The more uniform the terrain was, the fewer points were captured on the snow surface (and vice versa). The minimum distance between points in the horizontal plane was about 10cm at jumps and the maximum distance was 9.4m. On average 0.3 points were captured per 1m^2^. The course setting was captured either the day before or on the race day.

#### Forerunner center of mass trajectory

One forerunner trajectory per race was captured using a dynamic dGNSS. The dGNSS antenna (G5Ant-2AT1, Antcom, USA) was mounted on the helmet and a GPS/GLONASS dual frequency (L1/L2) receiver (Alpha-G3T, Javad, San Jose, CA, USA) was carried in a small cushioned backpack. Differential position solutions of the skier antenna trajectory were calculated at 50 Hz using the data from two base stations (antennas (GrAnt-G3T, Javad) and Alpha-G3T receivers (Javad)) and the geodetic post-processing software GrafNav (NovAtel Inc., Calgary, AL, Canada) [20].

The whole-body inclination of the forerunner was approximated using a virtual pendulum model, which was attached to the forerunner’s antenna position and driven by the accelerations acting on the antenna. An approximation of the center of mass position was reconstructed by intersection of the pendulum model with the digital model of the snow surface and a fraction of the distance between antenna and the intersection point of the pendulum with the snow surface [21,22].

### Parameter computation

#### Spatial reconstruction of course setting and terrain geomorphology

**Fig 2:**
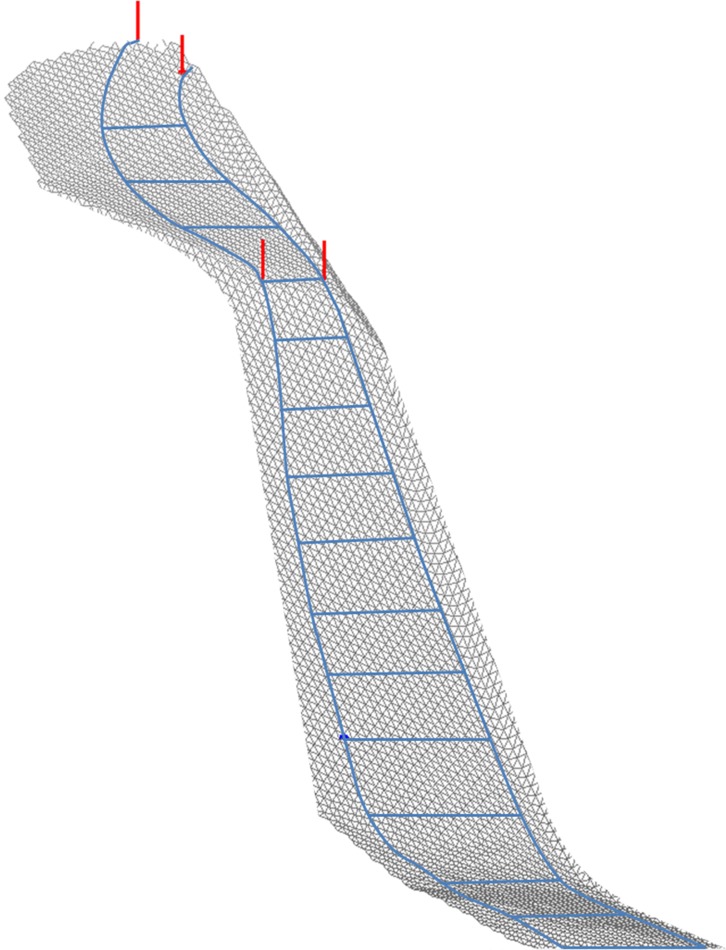
Illustration of the digital terrain model at the “Mausefalle” in the downhill race in Kitzbühel, Austria.

The captured points on the snow surface were triangulated using the method of Delaunay [23]. The triangulated surface was smoothed on a rectangular grid using bi-cubic spline functions [24,25] and represented as a digital terrain model (DTM) in MATLAB (MATLAB Inc., Natick, USA). The position of each gate was determined and represented in the DTM. An example of a DTM with course setting is shown in Fig. 2.

#### Course setting characteristics

The course setting was characterized, similar to the method used by Spörri et al.[16,26], by three distances: the gate distance, and the horizontal and the vertical gate distances. Gate distance is the linear distance (direct connection, not along the snow surface) from turning gate (i) to the previous turning gate (i−1). The horizontal distance is the distance between gate (i) and the normal projection of gate (i) on the vector from gate (i−1) to gate (i+1). The vertical distance is the distance from gate (i−1) to the projection of (i) onto the vector between (i−1) and (i+1). If two consecutive gates (i and i+1) defined one turn, both were projected on the vector from gate (i−1) and (i+2) to compute the horizontal gate distance. The gate with the largest horizontal gate distance was chosen to represent the turn. Gate distance and vertical gate distance were calculated for the respective gates. The course setting characteristics are illustrated in Fig. 1. The median gate distance and the median horizontal distance were used to calculate the median change in direction from gate (i−1) to gate (i) and gate (i+1).

#### Terrain inclination

**Fig 3:**
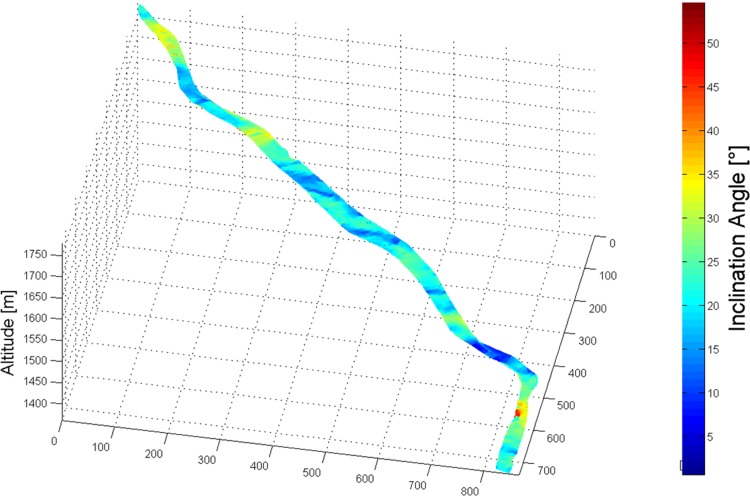
Illustration of the digital terrain model of the World Cup Giant Slalom slope “Chuenisbärgli” in Adelboden, Switzerland, colored according to terrain inclination.

To calculate terrain inclination the skier trajectory was projected normal to the DTM (P_S_). The terrain inclination angle was calculated as the angle between the gradient vector and the horizontal plane at every point of P_S_. Fig. 3 shows the digital terrain model of the World Cup GS slope in Adelboden, Switzerland, colored according to terrain inclination.

#### Terrain inclination in relation to course setting

**Fig 4:**
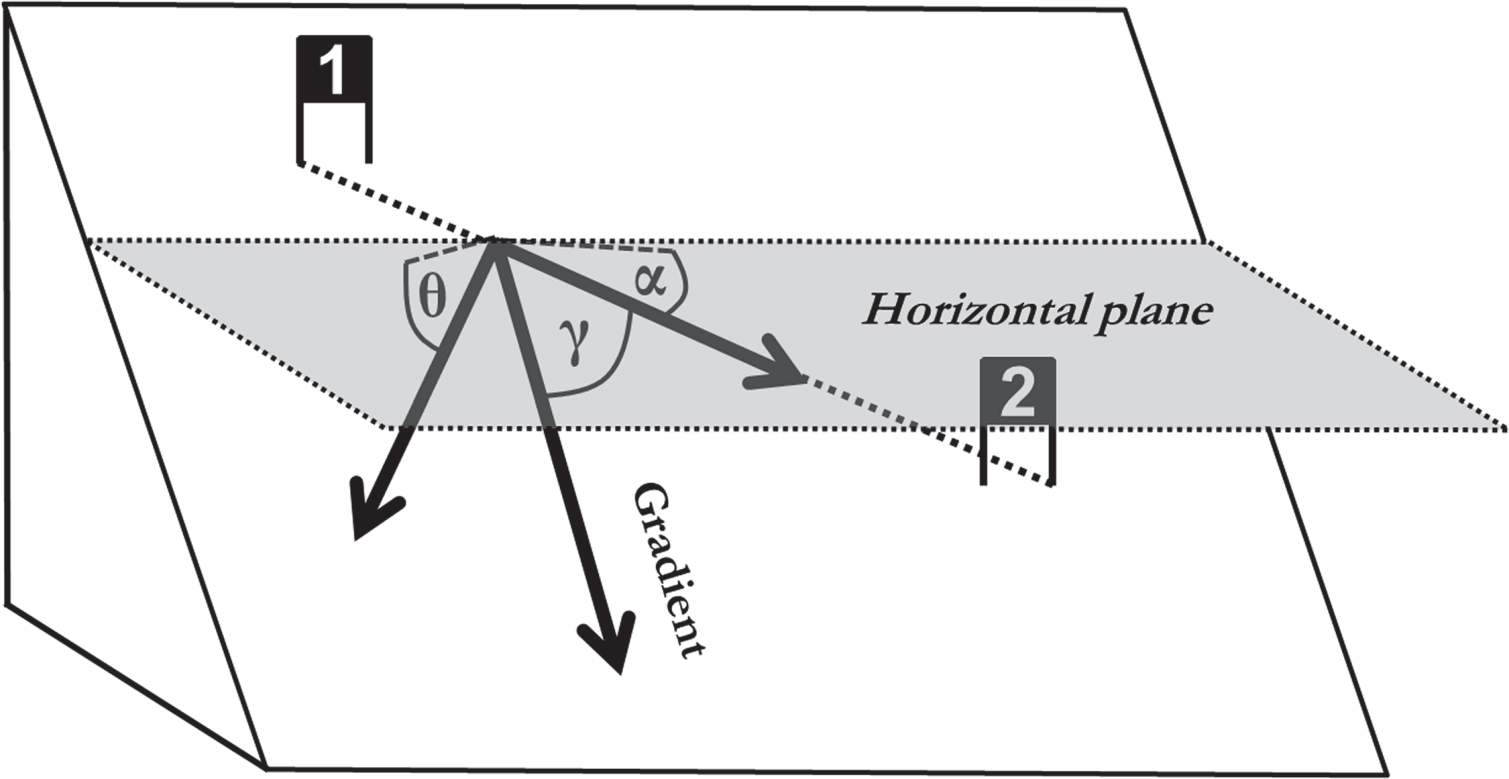
Illustration of the parameters describing the relationship between terrain and course setting: Terrain inclination in course direction (α), Angle between course direction and the gradient (β) and terrain inclination normal to course direction (γ).

The terrain inclination in the course direction (α) was calculated as the angle between the vector from gate (i) to gate (i+1) and the horizontal plane. The terrain inclination normal to the course direction (γ) was calculated as the terrain inclination in the direction normal to the vector from gate (i) to gate (i+1). The angle between course direction and the gradient (β) was calculated as the angle between the vector from gate (i) to gate (i+1) and the gradient. Terrain inclination in course direction (α) was calculated as a measure of how steep courses were set. Terrain inclination normal to course direction (γ) was calculated as a measure of how much the course was inclined in a lateral direction, causing a component of gravity to pull the skier in the lateral direction. The angle between course direction and the gradient (β) was calculated to show how much the course direction deviated from the gradient. The computation method for angles α, β and γ is illustrated in Fig. 4.

To assess the association between 1) the horizontal gate distance and the terrain inclination and 2) the gate distance and the terrain inclination, a Spearman correlation was used and significance was tested (α = 0.01).

#### Course Setting relative to terrain transitions

**Fig 5:**
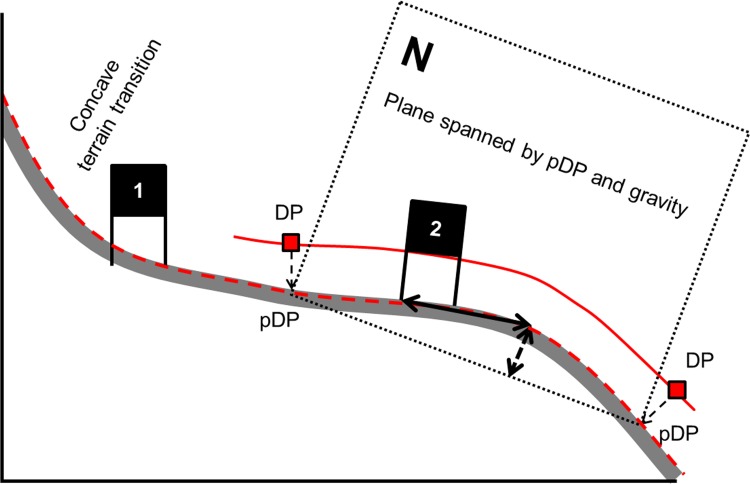
Illustration of terrain transition apex determination and the distance calculation between terrain transition apex and gate. The skier trajectory is shown in red with the deflection points of the trajectory (DP). The DPs were projected (pDP) normal onto the DTM (profile in dark grey) as well as the skier trajectory. The two pDPs and gravity span a plane. The terrain transition apex is where the arrows meet. The arrow with the dashed line represents the maximal distance to the vector between the pDPs. The solid arrow indicates the distance between the terrain apex and the gate (DTTG).

It was calculated how far gates were set from terrain transition (concave and convex) apices. Convex terrain transitions are terrain transitions where the terrain is bulging outward (bump). Concave terrain transitions are transitions where the terrain is hollowed inward (compression). The apex point of a terrain transition was calculated following a specific procedure: (1) The deflection points (DP) of the skier trajectory before and after the gate (i) were projected normal to the DTM (pDP) and a plane was spanned by the pDPs and gravity; (2) The projection of the skier trajectory onto the DTM (P_S_) was projected onto the plane spanned by pDP and gravity. The maximal distance of the projected P_S_ to the vector between the two pDP was defined as the terrain transition apex; (3) The distance from the terrain transition apex to the gate (i) was calculated and named (DTTG convex or DTTG concave). The calculation procedure for the distance from a terrain apex to the corresponding gate is illustrated in Fig. 5. The median distance and interquartile range (IQR) of the distance were calculated both for all data and for only the DTTG which were smaller than 10m for both convex and concave terrain transitions.

#### Terrain inclination relative to the skier trajectory

For terrain inclination, parameters were calculated relative to the course setting as shown, but also in relation to skier trajectory. The terrain inclination in the skier direction (parallel to P_S_), the terrain inclination in the direction normal to P_S,_ and the angle between P_S_ and the gradient were computed in the same way as the terrain inclination in the course direction (α), terrain inclination normal to course direction (γ) and the angle between course direction and the gradient (β) but using P_S_.

The instantaneous terrain inclination change along the skier trajectory was computed along the projection of the skier trajectory onto the DTM (P_S_). Terrain inclination was calculated for every position of P_S_ as the angle between the vector spanned by two consecutive points of P_S_ and the horizontal plane. The change in terrain inclination along P_S_ was computed both as the change in inclination per unit of distance travelled, and as the change in inclination per unit of time skiing. The change in terrain inclination per unit of distance travelled was calculated to characterize the terrain along the skier’s trajectory. The change in terrain inclination per unit of time travelled was calculated to characterize the rate at which skiers faced terrain inclination alterations along their path.

#### Skier Speed

Instantaneous center of mass position was derived from the dGNSS antenna position and the accelerations acting on it as described above. Speed was calculated as the time derivative of the center of mass position [8,22]. The mean speed for each turn was calculated as the mean of all speed measurements at 50 Hz along the trajectory between the transition from the previous turn to the respective turn and the transition to the upcoming turn. Turn transition was defined by the deflection point between two turns and was determined from the change in sign of the angular velocity [7,27].

#### Statistics

The number of gates analysed was 572 in GS, 210 in SG and 271 in DH. Normality of data was tested using a Lilliefors test (α = 0.05). Most parameters were found not to be normally distributed. Hence, non-parametric statistics were applied to all parameters. Median and IQR for all course setting and terrain parameters and skier speed were computed for all disciplines. The relative sizes of GS and SG compared to DH were computed from the medians of each discipline and were expressed as % of DH medians. The medians of the disciplines were tested for significant difference from each other for all parameters using an ANOVA Kruskal–Wallis test (α = 0.01), followed by a Friedman’s test (α = 0.01) when differences were found in the ANOVA. It was tested pairwise if the distributions of the three disciplines were statistically equal using a two-sample Kolmogorov–Smirnov test (α = 0.01). The distributions of terrain inclination change per distance and terrain inclination change in relation to time were assessed in detail for the largest 5% and smallest 5% values. For terrain inclination change per unit of distance it was determined whether the distributions were larger or smaller for all values larger than 1.3°/m and smaller than −1.2°/m, using a two-sample Kolmogorov–Smirnov test (α = 0.01). For terrain inclination change in relation to time it was determined whether the distributions were larger or smaller for all values larger than 19°/s and smaller than −21°/s using a two-sample Kolmogorov–Smirnov test (α = 0.01). The relationships between terrain inclination and gate distance and horizontal gate distance respectively were assessed with a linear model and the Spearman correlation coefficient was calculated to assess its strength. A Wilcoxon signed rank test (α = 0.05) was applied to test whether the distance from gate to terrain transition apex and terrain inclination changes were significantly different from zero. A Wilcoxon rank sum test and a two-sample Kolmogorov–Smirnov test (α = 0.01) were applied to determine whether the medians and distributions were different between: 1) terrain inclination in skier direction and terrain inclination in the course direction (α), 2) terrain inclination normal to skier direction and terrain inclination normal to the course direction, and 3) the angle between skier direction and gradient and the angle between course direction and gradient. It was determined whether distributions were similar between disciplines using a two sample Kolmogorov–Smirnov test (α = 0.01) and testing two disciplines consecutively. The relationships between terrain inclination and skier speed, gate distance and skier speed, and horizontal gate distance and skier speed were assessed with a linear model. Spearman’s rho correlation coefficient was also calculated and significance was tested for each discipline and for the data of all 3 disciplines pooled together (ALL). A multivariate linear model to explain skier speed by terrain inclination, gate distance and horizontal gate distance was developed for each discipline and for the data for ALL. The model was tested with an n-way ANOVA. The explained variance of the model (R^2^) and the significance of the model coefficients (p) were calculated. For each discipline, median speed and IQR were calculated using the speed measurements at 50 Hz from start to finish for each run.

## Results

### Course setting characteristics and terrain inclination

Median, IQR, significance of the difference between discipline medians and distributions and % of DH for GS and SG are presented in Table 1.

**Table 1:** Median, interquartile range (IQR) and significance level of the difference between discipline medians and distributions for all parameters, and percentage of DH for GS and SG.

	**Absolute Values (median and IQR)**			**Sign.**		**% of DH**	
	**GS**	**SG**	**DH**	**Median**	**Distr.**	**GS**	**SG**
Race length [m]	1437±65	2293±204	3499±501	*	*	41	66
Vertical drop [m]	407±23	598±38	859±112	*	*	47	69
# Gates / Race	53.8±3.4	44.3±3.3	41.5±6.5	1,2	*	130	106
Direction changes	51.2±3.5	40.8±4.0	-	1	1	-	-
Gate Distance [m]	26.24±2.25	49.48±5.69	79.10±37.27	*	*	33	63
Horizontal Gate Distance [m]	7.47±2.93	12.39±10.13	28.96±26.88	*	*	31	52
Vertical Gate Distance [m]	25.12±2.42	48.05±6.76	73.72±34.12	*	*	34	65
CV Gate Distance	0.09	0.11	0.47	-	-	18	24
CV Vertical Gate Distance	0.10	0.14	0.46	-	-	21	30
CV Horizontal Gate Distance	0.39	0.82	1.13	-	-	35	72
Terrain Inclination [°]	−17.8±7.0	−16.6±6.9	−13.6±7.7	*	*	131	121
DTTG Convex (<10m) [m]	1.61±4.79	−0.00±8.43	0.57±10.28			-	-
DTTG Concave (<10m) [m]	1.39±4.20	−0.92±6.67	0.00±5.07	1,2	1,2	-	-
Terrain Incl. in Course Direction [°]	−17.2±8.8	−16.2±6.4	−14.3±6.1	*	*	120	113
Terrain Incl. in Skier Direction [°]	−15.4±10.6	−14.3±11.0	−11.0±9.3	*	*	140	130
Terrain Incl. Normal to Course Dir. [°]	−8.2±6.0	−6.9±6.1	−8.9±8.5	2,3	*	92	77
Terrain Incl. Normal to Skier Dir. [°]	−6.3±6.2	−5.2±6.1	−5.9±7.4	2,3	*	107	86
Angle bw. Grad. & Course Dir.[°]	22.3±12.6	22.3±18.0	31.9±24.0	2,3	2,3	69	69
Angle bw. Grad. & Skier Dir. [°]	23.2±24.0	22.5±28.4	34.7±35.5	2,3	*	66	64
Terrain Inclination Change [°/m]	0.025±0.620	−0.009±0.436	0.008±0.468		*	-	-
Terrain Inclination Change [°/10s]	0.003±0.046	0.001±0.041	0.002±0.054		*	-	-

DH represents 100% for the relative measure. Differences between medians and distributions were significant between all disciplines if indicated with * and were significantly different between GS and SG when marked with 1, significantly different between GS and DH if marked with 2 and significantly different between SG and DH if marked with 3. If no parameter was significantly different the column is empty. Columns marked with—indicate that the measure was not calculated.

**Fig 6:**
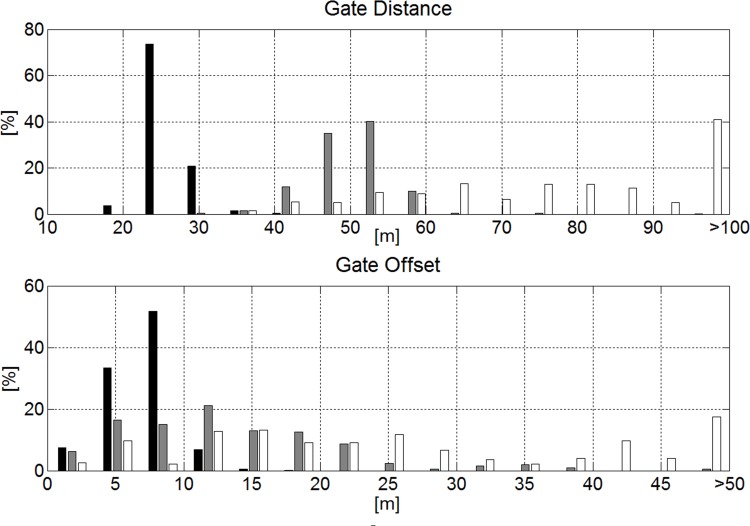
Course setting characteristics. The histograms illustrate the differences between disciplines for gate distance and horizontal gate distance. GS is shown in black, SG in gray and DH in white.

**Fig 7:**
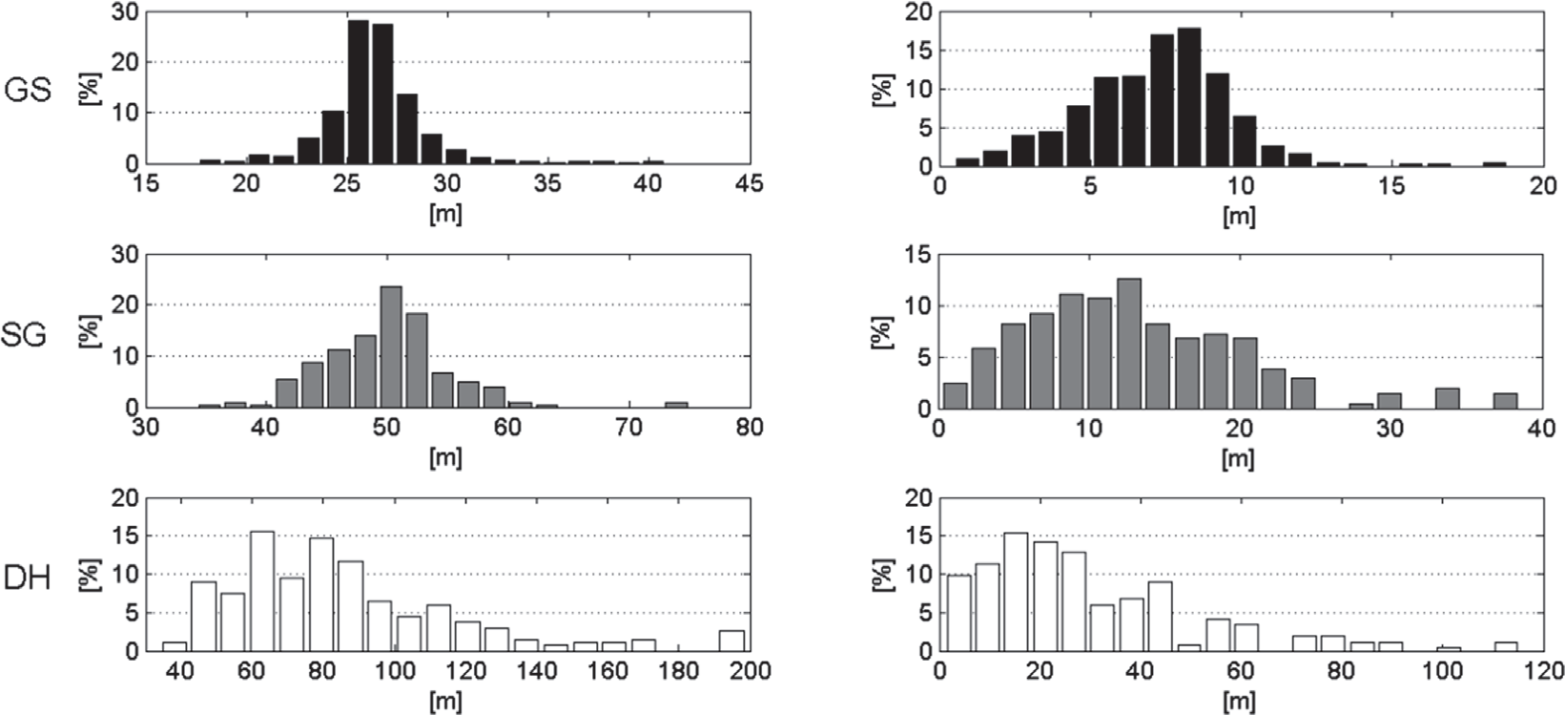
Course setting characteristics. The histograms represent each discipline alone. The left-hand column shows gate distance and the right-hand column indicates horizontal gate distance. GS is shown in black, SG in gray and DH in white.

**Fig 8:**
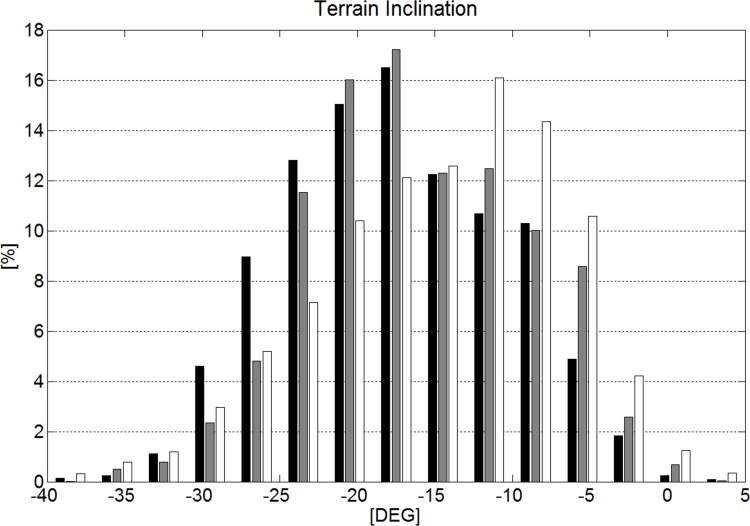
Histogram of the terrain inclination for the disciplines GS (black), SG (gray) and DH (white).

Race length, vertical drop and all gate distance parameters were significantly different and increased from GS to SG and DH. GS consisted of significantly more gates and direction changes per race than SG. The median change in direction of the course per turn was approximately equal for GS (31.5°) and SG (30.3°). The coefficient of variation (CV) was larger for horizontal gate distance than gate distance and vertical gate distance for all disciplines. In Figs. 6 and 7 the distributions between and within disciplines are shown for gate distance and horizontal gate distance. Terrain inclination was significantly different between all disciplines. Fig. 8 shows the histogram for terrain inclination.

**Fig 9:**
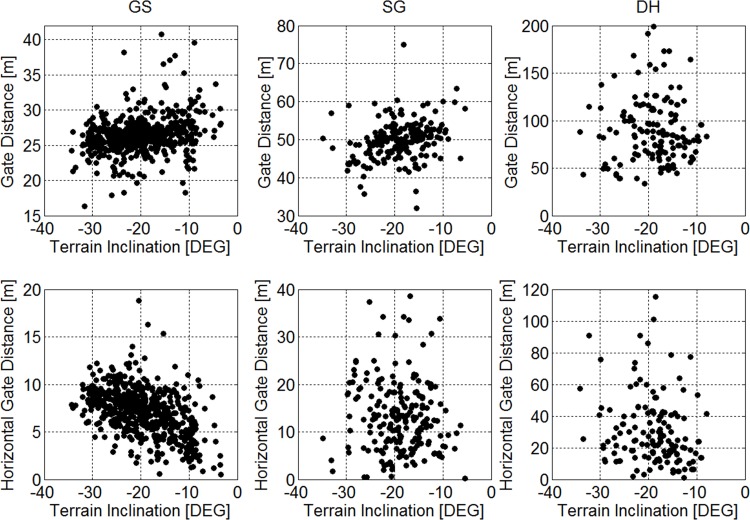
Scatterplots of the relationship between 1) terrain inclination and horizontal gate distance and 2) terrain inclination and gate distance for all disciplines.

Course Setting Relative to Terrain Inclination. Fig. 9 shows the course setting characteristics as a function of terrain inclination and Spearman’s Rho values, which were small for all relationships, but largest for horizontal gate distance in GS. There was a weak tendency to a shorter gate distance as the terrain became steeper in GS and SG (Table 2). In GS the horizontal gate distance increased with the steepness of the terrain inclination. Horizontal gate distance approximately doubled when terrain inclination increased from −10° to −30° in GS.

**Table 2:** Statistics of the relationship between terrain inclination and gate distances (gate distance and horizontal gate distance).

	**GS**	**SG**	**DH**
**Terrain Inclination—Gate Distance**			
Intersection	28.47	55.05	83.31
Inclination	0.11*	0.28*	−0.08
Spearman’s Rho	0.26	0.33	−0.04
**Terrain Inclination—Horizontal Gate Distance**			
Intersection	3.64	11.34	11.4
Inclination	−0.18*	−0.1	−0.92*
Spearman’s Rho	−0.47	−0.08	−0.31

* Significance level is 0.01

#### Terrain Inclination relative to Course Direction and Skier Trajectory

Table 1 reveals that terrain inclination in the skier’s direction and terrain inclination in the course direction (α) were steepest for GS, followed by SG and DH. Terrain inclination normal to skier direction, terrain inclination normal to course direction (γ), angle between skier direction and gradient and angle between course direction and gradient were larger for DH than GS and SG, while they were equal for GS and SG. Distributions were different for all parameters and disciplines except the angle between course direction and the gradient (β) for GS and SG.

**Fig 10:**
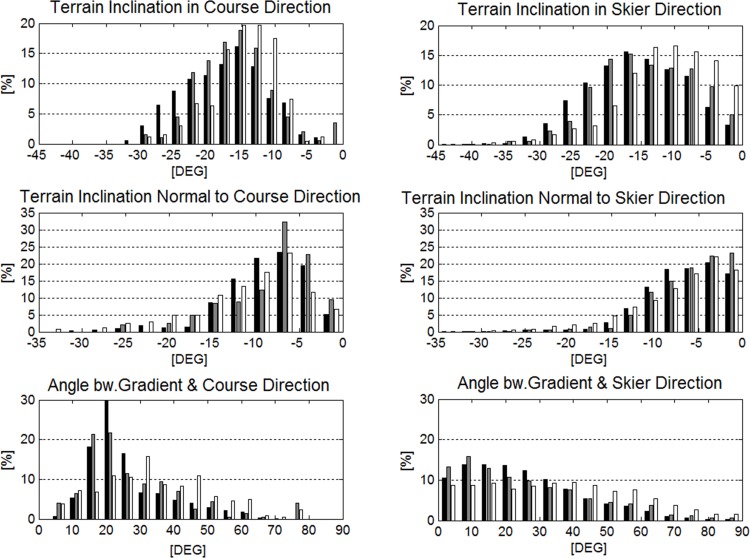
Terrain inclination features relative to course setting are presented in the left column. Terrain inclination features relative to the projection of the skier trajectory on the DTM are in the right column. The distributions for GS are shown in black, for SG in gray and for DH in white.

Terrain inclination in skier direction was significantly smaller than terrain inclination in course direction and terrain inclination normal to skier direction was significantly smaller than terrain inclination normal to course direction (γ) in all disciplines. The angle between skier direction and gradient was significantly smaller than the angle between course direction and gradient for GS only. The histograms of these parameters are illustrated in Fig. 10. All distributions were significantly different between skier and course direction.

**Fig 11:**
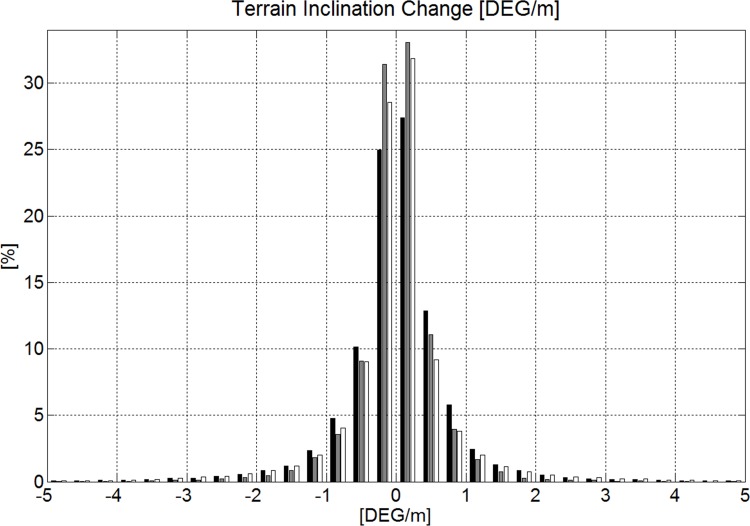
Terrain inclination change per meter of distance along the skier trajectory projected on the DTM. The disciplines are represented in black (GS), gray (SG) and white (DH).

**Fig 12:**
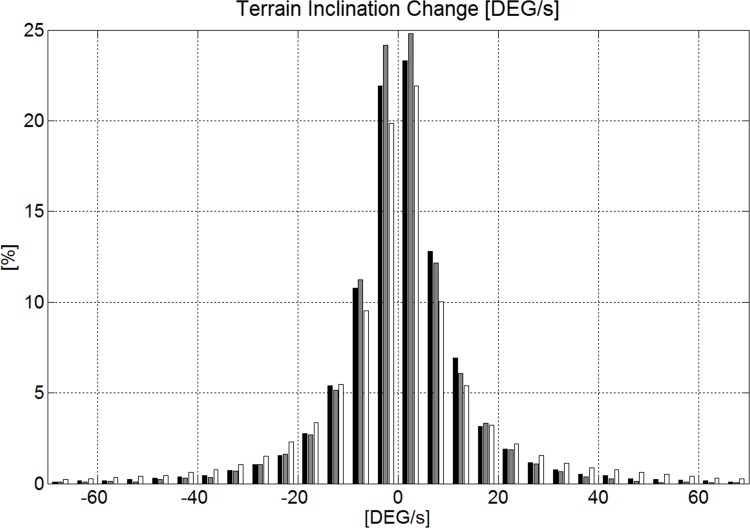
Terrain inclination change in relation to time expressed as degrees per second inclination change for skier trajectory projected on the DTM. The disciplines are represented in black (GS), gray (SG) and white (DH).

The median of terrain inclination change along P_S_ per meter skied and relative to time were not different from zero and are shown in histograms in Figs. 11 and 12. For the terrain inclination change along P_S_ per meter skied it was found that GS was significantly overrepresented compared to SG for values larger than 1.3°/m and values smaller than −1.2°/m, while GS and DH and SG and DH were not significantly different in their distribution of data in those ranges. For the terrain inclination change along P_S_ per second skied it was found that DH was significantly overrepresented compared to SG and GS for values larger than 19°/s and values smaller than −21°/s, while the distributions for GS and SG and were not significantly different from each other.

#### Course Setting Relative to Terrain Transitions

**Fig 13:**
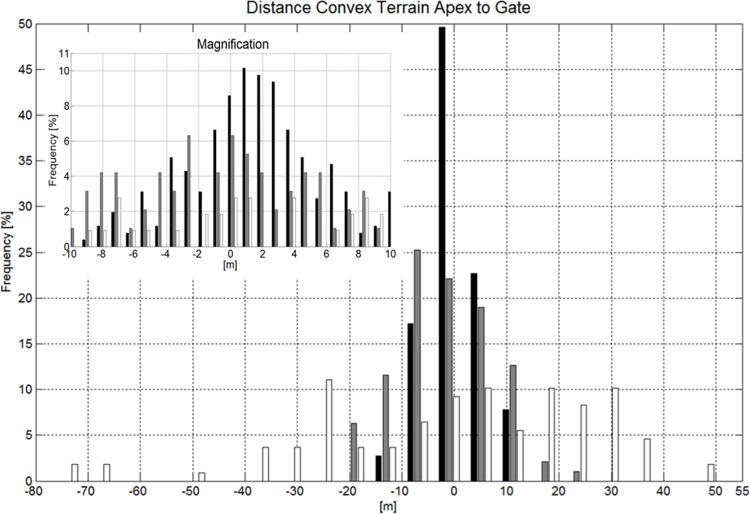
Histogram showing the distance distribution between convex terrain transition (ripple, wave) apex and gate positions for GS (black), SG (gray) and DH (white). A magnification of the histogram for the range from −10 to 10m with a higher resolution is shown in the upper left corner of the graph. A negative distance indicates that the gate is set ahead of the terrain transition apex, seen from the skier’s skiing direction.

**Fig 14:**
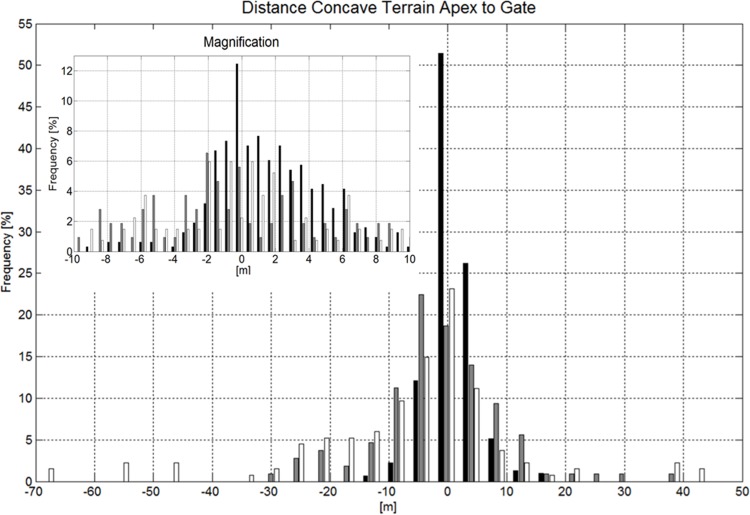
Histogram showing the distance distribution between concave terrain transition (compression) apex and gate positions for GS (black), SG (gray) and DH (white). A magnification of the histogram for the range from −10 to 10m with a higher resolution is shown in the upper left corner of the graph. A negative distance indicates that the gate is set ahead of the terrain transition apex, seen from the skier’s skiing direction.

Considering all gates, the distance between the gate and the apex of the terrain transition was significantly larger than 0 m and gates were set after terrain transitions for GS. In SG and DH the distances were not different from 0 m distance. In Figs. 13 and 14 the distances to terrain transition (convex and concave) apex are illustrated in histograms. If gates were set closer than 10m to the terrain transition in GS, the distances were also significantly different from zero and were on average set 1.61m after convex terrain transitions and 1.39m after concave terrain transitions. In SG and DH, distances were not different from zero if set closer than 10m to the terrain transition.

#### The Effect of Terrain Inclination and Course Setting on Skier Speed

**Fig 15:**
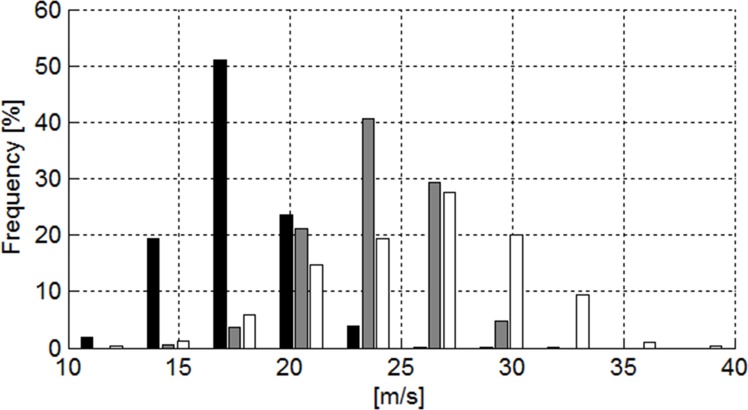
Histogram showing speed in m/s for GS (black), SG (gray) and DH (white).

Table 3 and Fig. 15 show that the median speed was highest for DH, followed by SG and GS. Medians and the distributions were significantly different between disciplines for speed.

**Table 3:** Statistics for speed.

**Median ± IQR**			**Sign.**		**% of DH**	
**GS**	**SG**	**DH**	**Median**	**Distr.**	**GS**	**SG**
17.75±2.30	23.82±2.70	25.61±4.34	*	*	69.28	92.98

Median, interquartile range (IQR) and significance level of the difference between discipline medians and distributions and percentage of DH for GS and SG. DH represents 100% for the relative measure. Differences between medians and distributions were significant between all disciplines as indicated with *.

**Fig 16:**
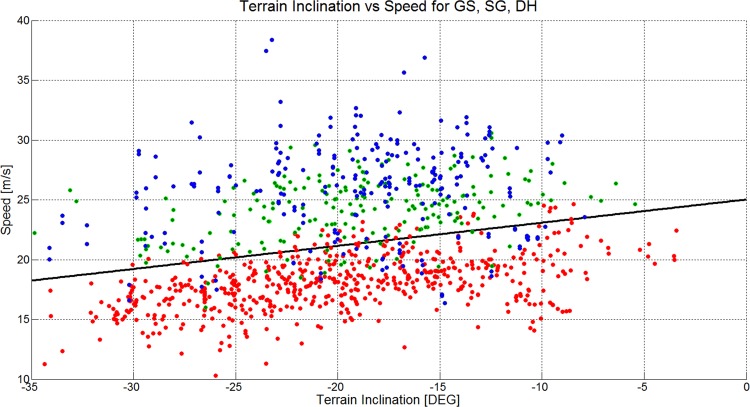
A scatterplot showing the terrain inclination versus skier speed for 1051 turns (GS: 572, SG: 210, DH: 271). The GS data is plotted in red, the SG data is plotted in green and the DH data is plotted in blue. The black line shows a linear regression model and the gray line a cubic model for the data of all disciplines.

**Fig 17:**
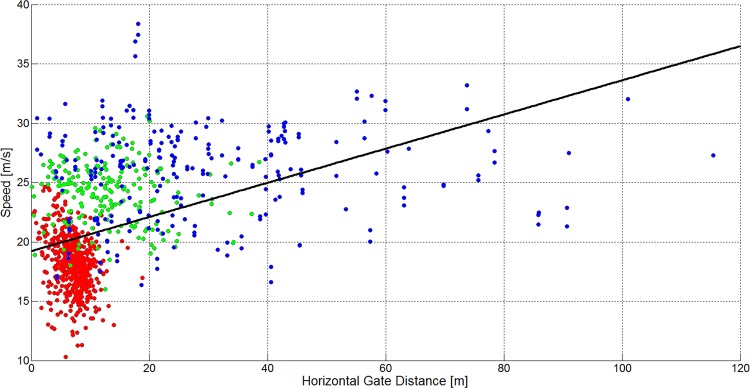
A scatterplot showing the horizontal gate distance versus skier speed for 1051 turns (GS: 572, SG: 210, DH: 271). The GS data is plotted in red, the SG data is plotted in green and the DH data is plotted in blue. The black line shows a linear regression model and the gray line a cubic model for the data of all disciplines.

**Fig 18:**
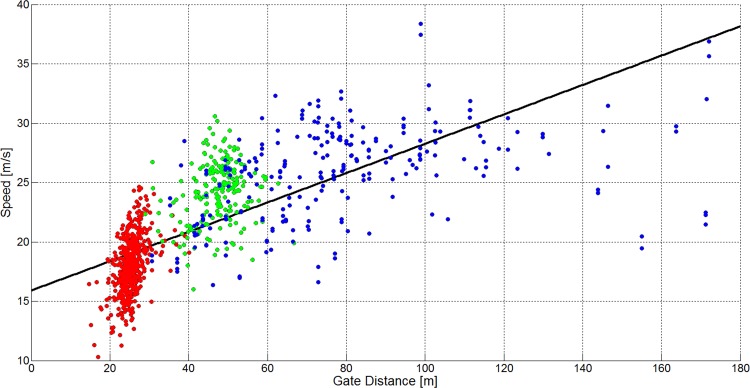
A scatterplot showing gate distance versus skier speed for 1051 turns (GS: 572, SG: 210, DH: 271). The GS data is plotted in red, the SG data is plotted in green and the DH data is plotted in blue. The black line shows a linear regression model and the gray line a cubic model for the data of all disciplines.

Fig. 16 and Table 4 show that skier speed increased significantly in GS, SG and for ALL as the terrain became flatter, but with different offsets (intersection) in each case. No significant relationship was found between terrain inclination and speed for DH. The linear model fitted best to GS, followed by SG, ALL and DH. Fig. 17 and Table 4 show that skier speed decreased significantly with increasing horizontal gate distance for GS and increased significantly with increasing horizontal gate distance for ALL. The relationship between horizontal gate distance and speed was not significantly different from zero for DH and SG. The linear model fit was best for ALL, followed by GS, DH and SG. Fig. 18 and Table 4 show that skier speed increased significantly with increasing gate distance for all three disciplines and ALL respectively. The linear model fitted best to ALL, followed by DH, GS and SG.

**Table 4:** Statistics of the linear relation between terrain inclination, gate distance, horizontal gate distance and speed respectively.

	**GS**	**SG**	**DH**	**ALL**
**Terrain Inclination—Skier Speed**				
Intersection	21.50	27.56	27.08	25.02
Inclination	0.17*	0.18*	0.07	0.19*
Spearman’s Rho	0.49	0.36	0.07	0.30
**Horizontal Gate Distance—Skier Speed**				
Intersection	20.24	24.71	24.97	19.21
Inclination	−0.31*	−0.04	0.03	0.14*
Spearman’s Rho	−0.34	−0.13	0.14	0.42
**Gate Distance—Skier Speed**				
Intersection	8.08	19.89	20.97	15.89
Inclination	0.40*	0.09*	0.06*	0.12*
Spearman’s Rho	0.46	0.21	0.52	0.81

The statistics for GS, SG and DH pooled together into one group are recorded as ALL.

* Significance level is 0.01

The model coefficients, their significance and explained variance (R^2^) for the multivariate linear model describing how terrain inclination, gate distance and horizontal gate distance are related to skier speed are shown in Table 5. The explained variance was largest for ALL, followed by GS, DH and SG. All coefficients were significant different from zero on a level of 0.05 for ALL and GS. The models for SG and DH included coefficients which were not significantly different from zero.

**Table 5:** Coefficients describing ANOVA of the multivariate linear model explaining skier speed using the factors terrain inclination, horizontal gate distance and gate distance for GS, SG, DH and the data of all disciplines pooled together (ALL).

**Discipline**	**R^2^**		**Constant**	**p1 (Terrain Inclination)**	**p2 (Gate Distance)**	**p3 (Horizontal Gate Distance)**
GS	0.34	Estimate	13.79	0.107	0.295	−0.189
		P	0.001	0.001	0.001	0.001
SG	0.14	Estimate	26.18	0.166	−0.032	−0.033
		p	0.001	0.001	0.366	0.148
DH	0.20	Estimate	21.98	0.053	0.006	−0.025
		p	0.001	0.191	0.001	0.062
ALL	0.57	Estimate	18.80	0.139	0.124	−0.038
		p	0.001	0.001	0.001	0.001

The form of the linear model is: Skier Speed = Constant + p1 Terrain Inclination + p2 Gate Distance + p3 Horizontal Gate Distance. Constant and p1 to p3 are the model coefficients. R^2^ describes the goodness of fit of the model to the data. p indicates the level of significance.

## Discussion

The main findings were that: 1) variability in horizontal gate distance (gate offset) was larger than in gate distance for all disciplines; 2) the horizontal gate distance increased with increasing terrain inclination in GS; 3) there was a slight trend towards shorter gate distances as the steepness of the terrain increased; 4) gates were usually set close to terrain transitions and; 5) terrain was flatter and was to a larger extent tilted in the lateral direction to the course direction in downhill compared to the other disciplines; 6) DH contained more extreme terrain inclination changes per unit time than the other disciplines; 7) Speed was significantly different between disciplines and increased as the terrain flattened in all disciplines except for DH; 8) In steep terrain, reduced skier speed was associated with increased horizontal gate distance and shorter gate distance in GS and reduced gate distance in SG and DH; 9) Across disciplines, skier speed was 57% explained by course setting characteristics and terrain inclination. The FIS competition regulations (Version July 16^th^, 2013) define a range of direction changes, respective to the number of gates. The number of direction changes is dependent on the altitude drop from start to finish and has to be in the range of 11–15% of the vertical drop in meters in GS. Applying the FIS regulation and the median vertical drop per race found in this study, the range for a typical GS race is 41 to 61 direction changes. The number of direction changes usually set is about in the middle of the range (51) determined by the FIS regulations for an average race. The given range is quite large and following the rules with respect to the number of direction changes is usually not a challenge for course setters. However, it might make sense to keep the range large, since course length and vertical drop vary between race locations and the rules have to cover all cases. Very steep and short races might challenge the lower limit. Flat and long courses might be in the upper range of allowed numbers of direction changes. Once it is established whether course setting can help prevent injuries, it might be useful to investigate whether more specific regulations, which take into account the steepness of the slope, could contribute to making courses safer. For male SG the regulations define the minimum number of gates and respective changes of direction at 7% of the vertical drop in meters. A minimum of 35 gates must be set as long as the vertical drop of the course is greater than 450m. The distance between the turning poles of two successive gates must be at least 25 m in normal situations. Using the data from this study, the numbers of direction changes are set close to the minimum given by the regulations. Hence, in order to reduce speed as a measure to potentially reduce injury risk, the current rules allow coaches to increase the number of direction changes from what is currently usual.

The coefficients of variation (CV) for gate distance and vertical gate distance were found to be small compared to the CV for the horizontal gate distance in all disciplines. Hence variability in course-setting geometry was regulated mainly by the horizontal gate distance, while gate distance and vertical gate distance were fairly constant. An explanation for the constant gate distances might be that course setters control the gate distance with distance measurement devices, probably to ensure that the gate distance regulation is followed and courses are set rhythmically. This study also showed a tendency for horizontal gate distance to increase with increasing terrain inclination in GS. In GS and SG, there was a weak tendency toward shorter gate distance when terrain inclination increased. These measures were probably taken to force the skier to turn more often and to a larger extent in steep sections in order to control speed when the component of gravity accelerating the skier was large due to the steep terrain. Controlling speed might be an important issue, since high speed is recognized as an important injury risk factor [2,3,7] and increased horizontal gate distance has been suggested as a measure to control speed [16] in GS. However, external force has also been suggested to be an injury risk factor [3,16] and force was found to vary with course setting [16,18]. Hence it might be worthwhile to investigate the effect of increased horizontal gate distance and/or shorter gate distances on skier speed and forces in different types of terrain.

It was found that gates were usually set close to terrain transitions in all disciplines. The reason for setting gates close to convex terrain transitions might be to ensure gates are fully visible. Avoiding gates which are hidden behind convex terrain transitions might help in avoiding accidents [4]. In GS, gates were usually set 1 to 2m behind the terrain transition apex, so that the gate was still visible to the athlete but could provide guidance about where the course was leading after the terrain transition. Other safety and competition considerations might also play a role. Turning in terrain transitions might increase the demands on skier technique (balance, timing) and strength compared to uniform terrain. Since turns are usually centered to the gate position [18] skiers’ balance might be challenged in convex terrain transitions because the snow surface tends to “disappear” under the skiers while they are turning. Further turning in convex terrain transitions might lead to additional unweighting and weighting of the skis and increased ground reaction forces at the spot where the skis are loaded. The combination of increased ground reaction force and edging of skis to turn might lead to additional damage to the snow surface [28], which has been associated with increased injury risk [3]. If turns are set close to concave terrain transitions (compressions), skiers might be challenged by increased forces, since the vertical component of the ground reaction force might increase as a result of the reduction in terrain inclination. Hence, it would be useful to investigate the effect of course setting at terrain transitions with respect to safety considerations.

The study showed that more than 85% of the time, terrain inclination change per meter of distance skied was less than 1° for all disciplines (Fig. 11) and hence alpine skiing courses are held on mostly uniform terrain regardless of the discipline. GS included a larger amount of extreme terrain inclination change per meter than GS and DH. In DH, rapid terrain inclination changes per unit time occurred to a larger extent than in GS and SG (Fig. 12). This might be due to the higher speed in DH [7]. Whether the abrupt terrain inclination changes (per unit time) in DH can be associated with the increased injury risk in DH [2,17] might be worth investigating.

Significant differences were found between disciplines in the extent to which terrain was tilted, not only in the lateral direction to the course direction but also in the direction the skier was skiing. It is probable that tilted terrain sets higher demands for balance, but it is unknown whether the increased amount of tilted terrain in speed disciplines can be associated with increased injury risk in the speed disciplines [2].

The medians and distributions of course and terrain characteristics and skier speed were different between the disciplines. This study investigated the relationship between course setting, terrain characteristics and skier speed in detail: Fig. 16 and Table 4 show a significant relationship between terrain inclination and skier speed for ALL, GS and SG but a non-significant relationship for DH. The component of gravity accelerating the skier is larger the steeper the terrain is. Consequently, if course setting is similar, it would be expected that skiers ski faster in steep terrain compared to flat terrain. However in GS, SG and ALL, it was found that speed was higher the flatter the terrain was. In GS, both the relationship between shorter gate distance and terrain inclination, and the relationship between increased horizontal gate distance and terrain inclination were strongest (Table 2). In SG, where gate distance, but not horizontal gate distance was significantly shorter in steeper terrain, speed was also significantly reduced. In DH, gate distance was slightly shorter in steep terrain, but speed was unchanged with terrain inclination. Hence, in steep terrain, speed seems to be controllable by course setting for GS and SG, but not for DH. When focusing on all disciplines (ALL), speed was found to significantly reduce with increasing steepness of the terrain. Furthermore, higher speed seems to be associated with longer gate distance and increased horizontal gate distance.

The hypothesis that skier speed is related to terrain inclination [19] and course setting [16,18] seems to be true for the relationship across the disciplines GS, SG and DH. A simple multivariate linear model describing speed as a function of terrain inclination, gate distance and horizontal gate distance explained 57% of the variance in skier speed (Table 5). The ANOVA of the multivariate linear model shows that terrain inclination, gate distance and horizontal gate distance explain a larger portion of the variance in speed for GS and across all disciplines (ALL) than in SG and DH. Hence, in SG and DH factors other than terrain inclination and course setting, such as increased air resistance [27,29–31], different equipment [32–34] or snow conditions [35–37], may influence skier speed as well. Fig. 16 to Fig. 18 show that the relationship between course setting and speed follows different characteristic modes for each disciplines. Hence, further studies should conduct a discipline-specific, in-depth analysis including a larger number of geomorphologic and skier mechanical factors. This might lead to a more profound understanding of how speed is regulated in competitive alpine skiing within the disciplines GS, SG and DH.

## Limitations

The forerunners capturing the data for this study skied slightly slower than the WC skiers in the same race. The time difference between our forerunners and the median of all skiers who completed the run was 2.4±2.1% for GS, 1.3±2.3% for SG and 5.3±1.2% for DH. The largest differences in DH occurred in gliding sections. Hence our data slightly underestimate the terrain inclination change measured in °/s compared to WC skiers.

To investigate the relationship between course setting and terrain inclination, the variables gate distance and horizontal gate distance were used. Gate distance and horizontal gate distance are not linearly independent. However, gate distance is the distance which is regulated by FIS competition rules. Moreover, it can be easily determined by distance measurement in daily coaching practice. Conversely, the variables horizontal and vertical distance gate distance exactly describe the gate setup, but are more complex to determine in field. As consequence, the variables gate distance and horizontal gate distance were chosen for this study, in order to meet both practical and scientific requirements. The effect of using gate distance and horizontal gate distance instead of horizontal and vertical gate distance on the explanatory power of the multiple linear model (Table 5) was found to be below 1%.

It may be that the chosen method to compare course setting and terrain geomorphology has limitations. Speed might be regulated by a series of gates and terrain characteristics over time. However, this is a topic which might be different for each discipline and should be assessed using an in-depth and discipline-specific approach in a future study.

## Conclusions

This study established a quantitative understanding of course and terrain characteristics, and their interrelation and effect on skier speed in male WC alpine skiing for the disciplines GS, SG and DH. This provides coaches with a source of information which should enable them to set training courses similar to WC competition courses. Furthermore, the findings of the current study may serve as a basis for future attempts assessing whether and how injury risk can be reduced by course setting.
