# Correction: Characterization of Course and Terrain and Their Effect on Skier Speed in World Cup Alpine Ski Racing

**DOI:** 10.1371/journal.pone.0128899

**Published:** 2015-05-20

**Authors:** Matthias Gilgien, Philip Crivelli, Jörg Spörri, Josef Kröll, Erich Müller

There is an error in Fig 4. The angles are incorrectly labeled. Please see the corrected Fig 4 here.

**Fig 4:**
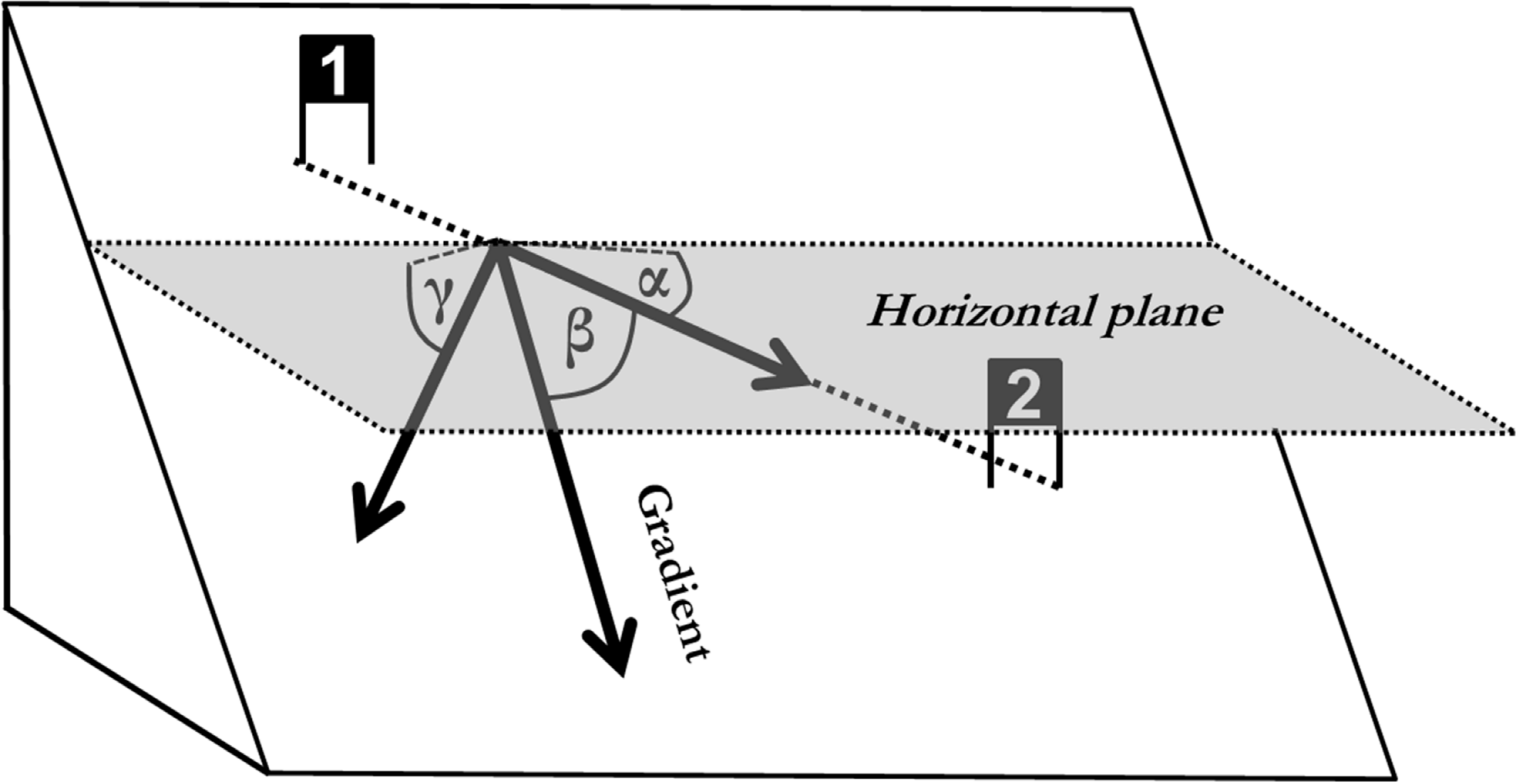
Illustration of the parameters describing the relationship between terrain and course setting: Terrain inclination in course direction (α), Angle between course direction and the gradient (β) and terrain inclination normal to course direction (γ).

There is an error in the caption for Fig 16. Please see the complete, correct Fig 16 caption here.

**Fig 16:**
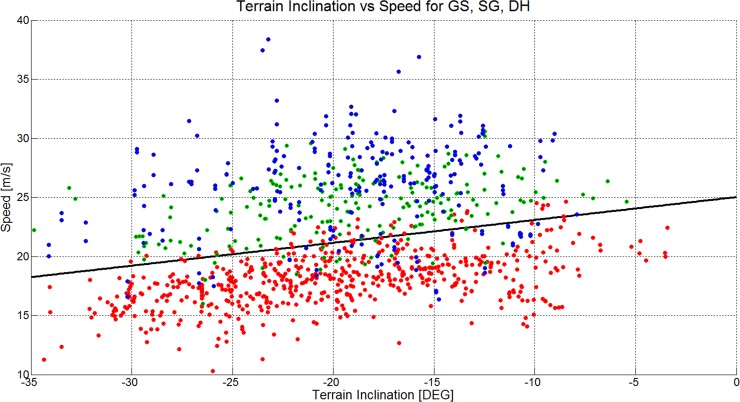
A scatterplot showing the terrain inclination versus skier speed for 1051 turns (GS: 572, SG: 210, DH: 271). The GS data is plotted in red, the SG data is plotted in green and the DH data is plotted in blue. The black line shows a linear regression model for the data of all disciplines.

There is an error in the caption for Fig 17. Please see the complete, correct Fig 17 caption here.

**Fig 17:**
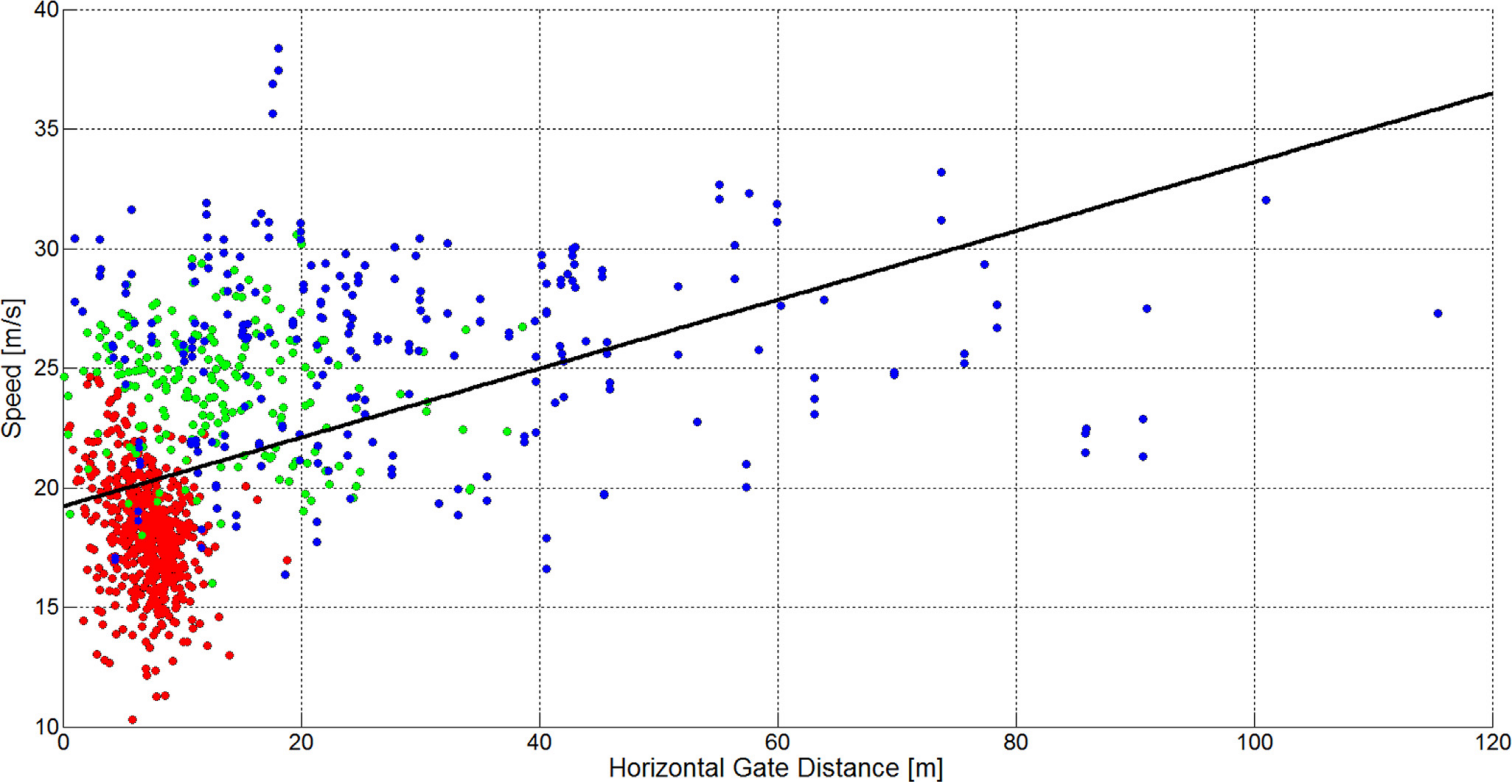
A scatterplot showing the horizontal gate distance versus skier speed for 1051 turns (GS: 572, SG: 210, DH: 271). The GS data is plotted in red, the SG data is plotted in green and the DH data is plotted in blue. The black line shows a linear regression model for the data of all disciplines.

There is an error in the caption for Fig 18. Please see the complete, correct Fig 18 caption here.

**Fig 18:**
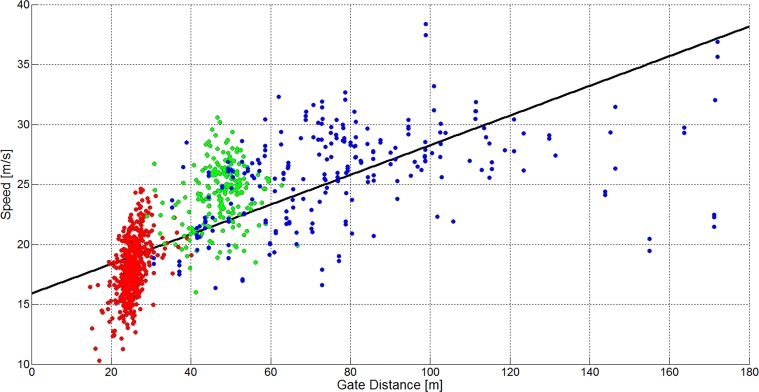
A scatterplot showing gate distance versus skier speed for 1051 turns (GS: 572, SG: 210, DH: 271). The GS data is plotted in red, the SG data is plotted in green and the DH data is plotted in blue. The black line shows a linear regression model for the data of all disciplines.
